# Supplementary material for: Powerful one-dimensional scan to detect heterotic quantitative trait loci
Source: Nat Commun. 2025 Nov 3;16:9697. doi: 10.1038/s41467-025-65563-9 (PMC12583706; doi:10.1038/s41467-025-65563-9)
Supplement: Supplementary file 2 — Reporting Summary [file 41467_2025_65563_MOESM2_ESM.pdf]

nature portfolio

Corresponding author(s): Yong Jiang, Jochen C. Reif

Last updated by author(s): Oct 7, 2025

# Reporting Summary

Nature Portfolio wishes to improve the reproducibility of the work that we publish. This form provides structure for consistency and transparency in reporting. For further information on Nature Portfolio policies, see our Editorial Policies and the Editorial Policy Checklist.

## Statistics

For all statistical analyses, confirm that the following items are present in the figure legend, table legend, main text, or Methods section.

| n/a | Confirmed | |
|---|---|---|
| ☐ | ☒ | The exact sample size (*n*) for each experimental group/condition, given as a discrete number and unit of measurement |
| ☐ | ☒ | A statement on whether measurements were taken from distinct samples or whether the same sample was measured repeatedly |
| ☐ | ☒ | The statistical test(s) used AND whether they are one- or two-sided<br>*Only common tests should be described solely by name; describe more complex techniques in the Methods section.* |
| ☐ | ☒ | A description of all covariates tested |
| ☐ | ☒ | A description of any assumptions or corrections, such as tests of normality and adjustment for multiple comparisons |
| ☐ | ☒ | A full description of the statistical parameters including central tendency (e.g. means) or other basic estimates (e.g. regression coefficient) AND variation (e.g. standard deviation) or associated estimates of uncertainty (e.g. confidence intervals) |
| ☐ | ☒ | For null hypothesis testing, the test statistic (e.g. *F*, *t*, *r*) with confidence intervals, effect sizes, degrees of freedom and *P* value noted<br>*Give P values as exact values whenever suitable.* |
| ☒ | ☐ | For Bayesian analysis, information on the choice of priors and Markov chain Monte Carlo settings |
| ☐ | ☒ | For hierarchical and complex designs, identification of the appropriate level for tests and full reporting of outcomes |
| ☒ | ☐ | Estimates of effect sizes (e.g. Cohen's *d*, Pearson's *r*), indicating how they were calculated |

*Our web collection on statistics for biologists contains articles on many of the points above.*

## Software and code

Policy information about availability of computer code

| | |
|---|---|
| Data collection | We didn't use any commercial, open source or custom code to collect data in this study. |
| Data analysis | A series of softwares were used to analyze the genomic data: SAMtools (v1.9), NovoSort (v3.06.05), plink (v 1.90b6.9), and Picard (v.2.21.9). Beagle (v 5.2) was used to impute missing values. Linear mixed models for analyzing the phenotypic data were implemented using the package ASReml-R (v4.1). Manhattan plots were generated using the R package CMplot57 (version 4.5.1), and circular plots were generated using the R package circlize58 (version 0.4.13). The hQTL-ODS model developed by us was implemented in R environment. All codes are available in GitHub (https://github.com/Ligl0226/hQTL-ODS/) and Zenodo (https://doi.org/10.5281/zenodo.17243742). |

For manuscripts utilizing custom algorithms or software that are central to the research but not yet described in published literature, software must be made available to editors and reviewers. We strongly encourage code deposition in a community repository (e.g. GitHub). See the Nature Portfolio guidelines for submitting code & software for further information.

## Data

Policy information about availability of data

All manuscripts must include a data availability statement. This statement should provide the following information, where applicable:

- Accession codes, unique identifiers, or web links for publicly available datasets
- A description of any restrictions on data availability
- For clinical datasets or third party data, please ensure that the statement adheres to our policy

Raw sequence data collected in this study have been deposited at the European Nucleotide Archive under Project PRJEB48738 (https://www.ebi.ac.uk/ena/browser/view/PRJEB48738, parental lines from Exp I, released in a previous study60) and PRJEB82869 (https://www.ebi.ac.uk/ena/browser/view/PRJEB82869, parental lines from Exp II and III). VCF files for whole WGS data are available from European Variation Archive under project PRJEB87554 (https://www.ebi.ac.uk/eva/?eva-study=PRJEB87554). The phenotypic data of all parents and hybrids, as well as the simulated data sets are provided in GitHub (https://github.com/Ligl0226/hQTL-ODS/). The genotypic and phenotypic data of the published maize dataset used in this study were accessed via the CNGBdb FTP public repository (https://ftp.cngb.org/pub/CNSA/data3/CNP0001565/zeamap/99_MaizegoResources/01_CUBIC_related/). Source data are provided with this paper (https://doi.org/10.6084/m9.figshare.30258319).

## Research involving human participants, their data, or biological material

Policy information about studies with human participants or human data. See also policy information about sex, gender (identity/presentation), and sexual orientation and race, ethnicity and racism.

| | |
|---|---|
| Reporting on sex and gender | Our study did not involve human participants, their data or biological material. |
| Reporting on race, ethnicity, or other socially relevant groupings | Our study did not involve human participants, their data or biological material. |
| Population characteristics | Our study did not involve human participants, their data or biological material. |
| Recruitment | Our study did not involve human participants, their data or biological material. |
| Ethics oversight | Our study did not involve human participants, their data or biological material. |

Note that full information on the approval of the study protocol must also be provided in the manuscript.

# Field-specific reporting

Please select the one below that is the best fit for your research. If you are not sure, read the appropriate sections before making your selection.

☒ Life sciences ☐ Behavioural & social sciences ☐ Ecological, evolutionary & environmental sciences

For a reference copy of the document with all sections, see nature.com/documents/nr-reporting-summary-flat.pdf

# Life sciences study design

All studies must disclose on these points even when the disclosure is negative.

| | |
|---|---|
| Sample size | Our study was based on 597 elite wheat lines and their 5,243 single-cross hybrid progenies. The parental lines were chosen to reflect a wide range of diversity in the Central European elite wheat population. The aim of using a dataset with large sample size was 1) to test the computational efficiency of our hQTL-ODS model, and 2) to maximize the statistical power of detecting heterotic QTL. |
| Data exclusions | Sequencing reads and single-nucleotide polymorphisms were filtered according to standard quality criteria (e.g. those used in ref. 60). This is to ensure that only high-quality marker data are used for subsequent analysis. Standard outlier tests (published in ref. 54) were performed for the phenotypic data and detected. To avoid potential problems in subsequent statistical analysis, outliers were pruned from the final datasets. |
| Replication | Generally, the estimated broad-sense heritability on the entry-mean basis was used to quantify the repeatability of quantitative traits, with a theoretical value of 1 meaning that measurements are perfectly repeatable. In our study, the estimated heritability for both traits were high (grain yield: > 0.75; heading date: > 0.90; see Table S3 for details). |
| Randomization | In each field trial, the alpha-lattice design was used to randomize the materials (genotypes) in order to correct for spatial variation. |
| Blinding | All sample names were coded in the field trials which ensures that scores were not influenced by knowing the identity of samples. |

# Reporting for specific materials, systems and methods

We require information from authors about some types of materials, experimental systems and methods used in many studies. Here, indicate whether each material, system or method listed is relevant to your study. If you are not sure if a list item applies to your research, read the appropriate section before selecting a response.

## Materials & experimental systems

| n/a | Involved in the study |
| --- | --- |
| ☒ | ☐ Antibodies |
| ☒ | ☐ Eukaryotic cell lines |
| ☒ | ☐ Palaeontology and archaeology |
| ☒ | ☐ Animals and other organisms |
| ☒ | ☐ Clinical data |
| ☒ | ☐ Dual use research of concern |
| ☐ | ☒ Plants |

## Methods

| n/a | Involved in the study |
| --- | --- |
| ☒ | ☐ ChIP-seq |
| ☒ | ☐ Flow cytometry |
| ☒ | ☐ MRI-based neuroimaging |

# Dual use research of concern

Policy information about dual use research of concern

## Hazards

Could the accidental, deliberate or reckless misuse of agents or technologies generated in the work, or the application of information presented in the manuscript, pose a threat to:

| No | Yes |
| --- | --- |
| ☒ | ☐ Public health |
| ☒ | ☐ National security |
| ☒ | ☐ Crops and/or livestock |
| ☒ | ☐ Ecosystems |
| ☒ | ☐ Any other significant area |

## Experiments of concern

Does the work involve any of these experiments of concern:

| No | Yes |
| --- | --- |
| ☒ | ☐ Demonstrate how to render a vaccine ineffective |
| ☒ | ☐ Confer resistance to therapeutically useful antibiotics or antiviral agents |
| ☒ | ☐ Enhance the virulence of a pathogen or render a nonpathogen virulent |
| ☒ | ☐ Increase transmissibility of a pathogen |
| ☒ | ☐ Alter the host range of a pathogen |
| ☒ | ☐ Enable evasion of diagnostic/detection modalities |
| ☒ | ☐ Enable the weaponization of a biological agent or toxin |
| ☒ | ☐ Any other potentially harmful combination of experiments and agents |

# Plants

| | |
| --- | --- |
| Seed stocks | The elite parental lines were provided by breeding companies. Details were described in previous studies (Boeven et al. 2020, Zhao et al. 2021). |
| Novel plant genotypes | The single-cross hybrid progenies were produced by using chemical hybridization agents. |
| Authentication | The sterility of the female parental lines after chemical hybridization agent application was checked by bagging one to three plants. |
