# Supplementary material for: Powerful one-dimensional scan to detect heterotic quantitative trait loci
Source: Nat Commun. 2025 Nov 3;16:9697. doi: 10.1038/s41467-025-65563-9 (PMC12583706; doi:10.1038/s41467-025-65563-9)
Supplement: Supplementary file 3 — Description of Additional Supplementary Files [file 41467_2025_65563_MOESM3_ESM.pdf]

Description of additional Supplementary Files

File name: Supplementary Data 1

Description: Overview of the number of genotypes within each experimental series and the number of phenotyping environments.

File name: Supplementary Data 2

Description: Summary of parameters in the five simulated scenarios.

File name: Supplementary Data 3

Description: The association between the hQTL detection power and heterozygosity.

File name: Supplementary Data 4

Description: Overview of the phenotype data and summary statistics within the three experimental series and the integrated panel.

File name: Supplementary Data 5

Description: Summary of whole genome resequencing and a comparison with 90k SNP chip data.

File name: Supplementary Data 6

Description: Number of overlapping genotypes bridging the three experimental series and the phenotypic correlation between different experimental series.

File name: Supplementary Data 7

Description: Variance components and broad-sense heritability of midparent heterosis for heading date and grain yield.

File name: Supplementary Data 8

Description: Heterotic QTL for grain yield heterosis detected by hQTL-ODS in integrated panel.

File name: Supplementary Data 9

Description: Dominance QTL for grain yield detected in integrated panel.

File name: Supplementary Data 10

Description: Heterotic QTL for heading date heterosis detected by hQTL-ODS in integrated panel.

File name: Supplementary Data 11

Description: Dominance QTL for heading date detected in integrated panel.
